# Urinary Tract Infections

**DOI:** 10.3390/antibiotics3030375

**Published:** 2014-08-14

**Authors:** Truls E. Bjerklund Johansen, Kurt G. Naber

**Affiliations:** 1 Urology Department, Oslo University Hospital, 0424 Oslo, Norway; 2 Urology Department, Technical University of Munich, Munich 81675, Germany

Urinary tract infections (UTI) are among the most frequently acquired infections in the community, but also in hospitals and other health care institutions, causing a huge amount of antibiotic consumption. During the last decade we have seen significant changes in the field of urinary tract infections regarding causative pathogens and antibiotic treatment calling for an update of current trends.

The worldwide increase of uropathogens resistant to former first line antibiotics, such as cotrimoxazole, fluoroquinolones and cephalosporins, has had detrimental consequences not only for treatment but also for prophylaxis of infectious complications after urological interventions. A paradigm shift concerning asymptomatic bacteriuria has had a great impact on the definition and management of UTIs today [1,2,3,4].

For uncomplicated lower UTI, such as acute cystitis in otherwise healthy women, not only a revival of old (oral) antibiotics, such as fosfomycin trometamol, pivmecillinam, nitrofurantoin, can be observed in many guidelines [5,6,7], but even a non-antimicrobial measure has been tested in a pilot study [8]. It will therefore be interesting to see the results of forthcoming phase III studies and whether antibiotic therapy could at least be partially replaced. For prophylaxis of recurrent episodes of uncomplicated UTI, non-antimicrobial measures are already preferred and antimicrobial prophylaxis is only recommended as a last resort [7].

However, for complicated, nosocomial and severe UTI including pyelonephritis, antibiotic therapy will still be a corner stone in combination with treatment of the underlying complicating conditions. Unfortunately, there are few new antimicrobial drugs in the pipelines of pharmaceutical companies with prospects to overcome the problem of multi and extended drug resistant uropathogens [9].

Although the classical distinction between uncomplicated and complicated UTI is still valid in principle, the different criteria to be considered are so heterogeneous, that a better (phenotypical) subclassification might be helpful, as proposed by the European Section of Infection of Urology (ESIU) of the European Association of Urology (EAU) [10].

In consideration of so many new aspects related to optimal management of UTI, it has been our pleasure to edit a joint presentation of the results from different research groups in one special scientific publication challenging established as well as new scientific approaches to improve prophylaxis and treatment of patients suffering from UTI.
